# Genetic architecture of soybean tolerance to off-target dicamba

**DOI:** 10.3389/fpls.2023.1230068

**Published:** 2023-10-09

**Authors:** Caio Canella Vieira, Jing Zhou, Diego Jarquin, Jianfeng Zhou, Brian Diers, Dean E. Riechers, Henry T. Nguyen, Grover Shannon

**Affiliations:** ^1^ Crop, Soil, and Environmental Sciences, University of Arkansas, Fayetteville, AR, United States; ^2^ Biological Systems Engineering, University of Wisconsin-Madison, Madison, WI, United States; ^3^ Agronomy Department, University of Florida, Gainesville, FL, United States; ^4^ Division of Plant Science and Technology, University of Missouri, Columbia, MO, United States; ^5^ Department of Crop Sciences, University of Illinois, Urbana, IL, United States

**Keywords:** soybean, genome-wide association studies, machine learning, plant breeding, dicamba, abiotic stress

## Abstract

The adoption of dicamba-tolerant (DT) soybean in the United States resulted in extensive off-target dicamba damage to non-DT vegetation across soybean-producing states. Although soybeans are highly sensitive to dicamba, the intensity of observed symptoms and yield losses are affected by the genetic background of genotypes. Thus, the objective of this study was to detect novel marker-trait associations and expand on previously identified genomic regions related to soybean response to off-target dicamba. A total of 551 non-DT advanced breeding lines derived from 232 unique bi-parental populations were phenotyped for off-target dicamba across nine environments for three years. Breeding lines were genotyped using the Illumina Infinium BARCSoySNP6K BeadChip. Filtered SNPs were included as predictors in Random Forest (RF) and Support Vector Machine (SVM) models in a forward stepwise selection loop to identify the combination of SNPs yielding the highest classification accuracy. Both RF and SVM models yielded high classification accuracies (0.76 and 0.79, respectively) with minor extreme misclassifications (observed tolerant predicted as susceptible, and vice-versa). Eight genomic regions associated with off-target dicamba tolerance were identified on chromosomes 6 [Linkage Group (LG) C2], 8 (LG A2), 9 (LG K), 10 (LG O), and 19 (LG L). Although the genetic architecture of tolerance is complex, high classification accuracies were obtained when including the major effect SNP identified on chromosome 6 as the sole predictor. In addition, candidate genes with annotated functions associated with phases II (conjugation of hydroxylated herbicides to endogenous sugar molecules) and III (transportation of herbicide conjugates into the vacuole) of herbicide detoxification in plants were co-localized with significant markers within each genomic region. Genomic prediction models, as reported in this study, can greatly facilitate the identification of genotypes with superior tolerance to off-target dicamba.

## 1 Introduction

With over 95% of the soybean [*Glycine max* (L.) Merr.] acreage grown with genetically-engineered herbicide-tolerant cultivars in the United States, dicamba (3,6-dichloro-2-methoxybenzoic acid)-tolerant (DT) soybean seeds are planted in nearly 22.3 million hectares each year (Tindall et al., 2021; USDA Economic Research Service, 2022). The widespread adoption of DT soybean since 2016 resulted in extensive off-target damage to non-DT soybean and other dicots plants (Bradley, 2017; Bradley, 2018; Wechsler et al., 2019; Chism et al., 2020; Wagman et al., 2020). From 2016 to 2021, the Environmental Protection Agency (EPA) recorded more than 10,500 reports of dicamba-related injuries in non-DT vegetation across 29 of the 34 states where the over-the-top use of dicamba is authorized (Echeverria, 2020; Tindall et al., 2021). Due to its high vapor pressure, dicamba is prone to increased occurrences of off-target movement to nearby fields (Behrens and Lueschen, 1979; Egan and Mortensen, 2012). Environmental conditions consisting of high temperatures and relative humidity (Egan and Mortensen, 2012), and lower soil pH (Oseland et al., 2020), often observed in soybean-producing regions during the growing season, can exacerbate off-target movement. Based on market research and aggregated sales data, 60% of the acreage planted with DT soybean was treated at least once with dicamba. Thus, there were up to 8.9 million hectares grown with DT soybean seeds not for the herbicide-based weed management system but as protection against unintentional off-target dicamba exposure (Tindall et al., 2021).

As a growth regulator herbicide, dicamba is a synthetic auxin that triggers fast and uncontrolled growth of the stems, petioles, and leaves resulting in the death of sensitive dicots (Grossmann, 2010). Soybean is highly sensitive to dicamba. Symptoms of dicamba exposure include crinkling and cupping of immature leaves, decreased plant height, apical meristem death, abnormal pod formation, and reduced grain yield (Weidenhamer et al., 1989; Andersen et al., 2004; Grossmann, 2010; Kniss, 2018; Canella Vieira et al., 2022b). Timing, dosage, frequency, and duration of exposure have been shown to affect the severity of the symptoms. For instance, soybean is far more sensitive to dicamba exposure at the early reproductive stage relative to the vegetative stage (Egan et al., 2014; Solomon and Bradley, 2014; Soltani et al., 2016; Kniss, 2018). Recently, different genetic backgrounds were reported to also influence the intensity of symptomology resulting from off-target dicamba in soybean (Canella Vieira et al., 2022b). That study reported differential responses of conventional soybean genotypes to off-target dicamba, where certain genetic backgrounds showed consistently superior responses with minimal symptoms and yield losses under prolonged off-target dicamba exposure (Canella Vieira et al., 2022b).

Genome-wide association studies (GWAS) are performed to detect significant associations between a trait of interest and molecular markers using linear regression models (Yu et al., 2006; Hwang et al., 2014; Vuong et al., 2015; Zhang et al., 2015) as well as machine and deep learning algorithms (Liu et al., 2019; Yoosefzadeh-Najafabadi et al., 2021; Canella Vieira et al., 2022c; Yoosefzadeh-Najafabadi et al., 2022). Using a panel of genetically diverse soybean accessions, significant associations were reported between off-target dicamba response and single nucleotide polymorphisms (SNPs) on chromosomes 10 [Linkage Group (LG) O], 11 (LG B1), 15 (LG E), 18 (LG G), and 19 (LG L) (Canella Vieira et al., 2022a). Interestingly, the identified associations are located in genomic regions nearby genes with annotated functions consisting of various phases of herbicide detoxification in plants (Canella Vieira et al., 2022a). This includes oxidation or hydrolysis by cytochrome P450s and carboxylesterases, respectively (Phase I) (Kreuz et al., 1996; Barrett, 2000), uridine diphosphate (UDP)-dependent glycosyltransferases conjugation of hydroxylated herbicides to endogenous sugar molecules (Phase II) (Riechers et al., 2010), and transportation of phase II-conjugated herbicide by multidrug resistance proteins (MRPs) into the vacuole (Phase III) (Riechers et al., 2010).

Thus, the purpose of this study was to conduct GWAS to identify novel marker-trait associations and expand on previously identified genomic regions in a new population with different genetic backgrounds than Canella Vieira et al. (2022a). A machine learning (ML)-GWAS pipeline incorporating a supervised feature dimension reduction based on Variable Importance in Projection (VIP) and classification algorithms was implemented to identify the combination of SNPs that provided the highest classification accuracy for off-target dicamba response. Identification and characterization of the genetic architecture of soybean tolerance to off-target dicamba and the development of non-DT tolerant genotypes may sustain the production and adoption of other genetically engineered herbicide-tolerant soybean production systems in regions severely affected by off-target dicamba exposure, as well as the expanding niche markets based of organic and conventional soybean.

## 2 Materials and methods

### 2.1 Plant material and genomic data

Soybean genotypes consisted of 551 non-DT advanced breeding lines derived from 232 unique bi-parental populations. In addition, 18 commercial cultivars [14 DT and four non-DT glyphosate [(*N*-(phosphonomethyl)glycine)]-tolerant (GT)] were included in the study as controls to confirm the presence of off-target dicamba exposure and assess the homogeneity of off-target dicamba distribution (Canella Vieira et al., 2022b). In 2019, plant materials consisted of 210 advanced breeding lines, three GT commercial cultivars, and seven DT commercial cultivars. In 2020, plant materials consisted of 204 advanced breeding lines, three GT commercial cultivars, and six DT commercial cultivars. In 2021, 209 advanced breeding lines, three GT commercial cultivars, and 11 DT commercial cultivars were evaluated. In the study, some overlapping of genotypes across years was observed, hence the total number of genotypes evaluated across environments included more than 551 advanced breeding lines and 18 commercial cultivars. The maturity group (MG) of genotypes ranged from 4-early to mid-5. MG was noted as the number of days after August 1^st^ when 95% of pods on the main stem had reached mature brown color (Fehr and Caviness, 1977). Relative maturity (RM) was calculated as days earlier or later than reference controls and was used to assign MG, where 4-early = 4.0 to 4.3 (88 genotypes), mid-4 = 4.4 to 4.6 (127 genotypes), 4-late = 4.7 to 4.9 (171 genotypes), 5-early = 5.0 to 5.3 (138 genotypes), and mid-5 = 5.4 to 5.6 (27 genotypes) (Canella Vieira et al., 2022b). All soybean breeding lines were genotyped using the Illumina Infinium BARCSoySNP6K BeadChip (Song et al., 2020) at the USDA-ARS Soybean Genomics and Improvement Laboratory (Beltsville, MD). A total of 4,970 SNPs were obtained after filtering based on minor allele frequency (MAF)< 0.05.

### 2.2 Field experiments and data collection

Nine environments (combination of location, field, and year) under prolonged off-target dicamba exposure were used to conduct field experiments for three years (2019-2021) in Portageville, MO (36°23’44.2”N lat; 89°36’52.3”W long). Genotypes were distributed in field trials based on MG. Each field trial was arranged in a three-replicate randomized complete block design where each plot consisted of four 3.66 m long rows spaced 0.76 m apart. The homogeneity of off-target dicamba exposure was assessed and confirmed using an inhomogeneous Poisson marked point process (Daley and Vere-Jones, 2003) based on the spatial distribution of the relative yield performance between GT and nearby DT commercial cultivars (Canella Vieira et al., 2022b).

Soybean genotypes were visually assessed for off-target dicamba damage on a 1 to 4 scale with 0.5 increments between R1 and R3 (Fehr et al., 1971). As described by Canella Vieira et al. (2022b), a damage rating of 1 showed symptomology equivalent to the DT control with none to minimal visual dicamba damage symptoms; a damage rating of 2 showed moderate tolerance with modest cupping of the immature leaves without effect on canopy coverage and vegetative growth; a damage rating of 3 showed intensified cupping of the immature leaves and moderate reduction in canopy coverage and vegetative growth, and a rating of 4 showed extreme damage symptomology including drastic cupping of the immature leaves and acute reduction in canopy coverage and vegetative growth (Canella Vieira et al., 2022b). The consistency and reliability of scores across and within environments were confirmed using Pearson’s correlation coefficients and Cronbach’s alpha (Cronbach, 1951), respectively, as reported by Canella Vieira et al. (2022b).

Damage ratings were adjusted across environments using the function ‘*ls_means*’ of the R (R Core Team, 2023) package ‘*lmerTest*’ (Kuznetsova et al., 2017). A mixed-effects linear model including the fixed effect of ‘genotype’, the random interaction between ‘genotype’ and ‘environment’ (G×E), the random effect of ‘environment’, and the nested random effect of ‘replication’ within ‘environment’ was fitted using the package ‘*lme4’* (Bates et al., 2015). Genotypes were classified into three categories based on the adjusted off-target dicamba damage: tolerant when damage scores ≤ 2, moderate > 2, ≤3, and susceptible >3.

### 2.3 Genome-wide association study

Two linear regression-based models were utilized to conduct GWAS, including the Fixed and Random Model Circulating Probability Unification (FarmCPU) (Liu et al., 2016) and the Bayesian-information and Linkage-disequilibrium Iteratively Nested Keyway (BLINK) (Huang et al., 2019). In addition, one ML-GWAS pipeline incorporating feature dimension reduction and classification algorithms was implemented. In summary, FarmCPU maximizes the advantages of mixed linear models and stepwise regression by using them iteratively. It substitutes kinship with a set of molecular markers fitted as fixed effects that are tested one at a time across the genome. The molecular markers are optimized in a restricted maximum likelihood method in a mixed linear model with variance and covariance defined by the set of pre-selected molecular markers, reducing the risk of model overfitting (Liu et al., 2016). BLINK is an improved version of FarmCPU that discards the assumption that genes associated with a trait are evenly spread across the genome. It replaces the restricted maximum likelihood method with Bayesian Information Content (BIC) to improve computing speed (Huang et al., 2019). Both FarmCPU and BLINK models were conducted using the R (R Core Team, 2023) package “*GAPIT*” (Lipka et al., 2012).

### 2.4 Feature selection and machine learning classification algorithms

**Figure 1:**
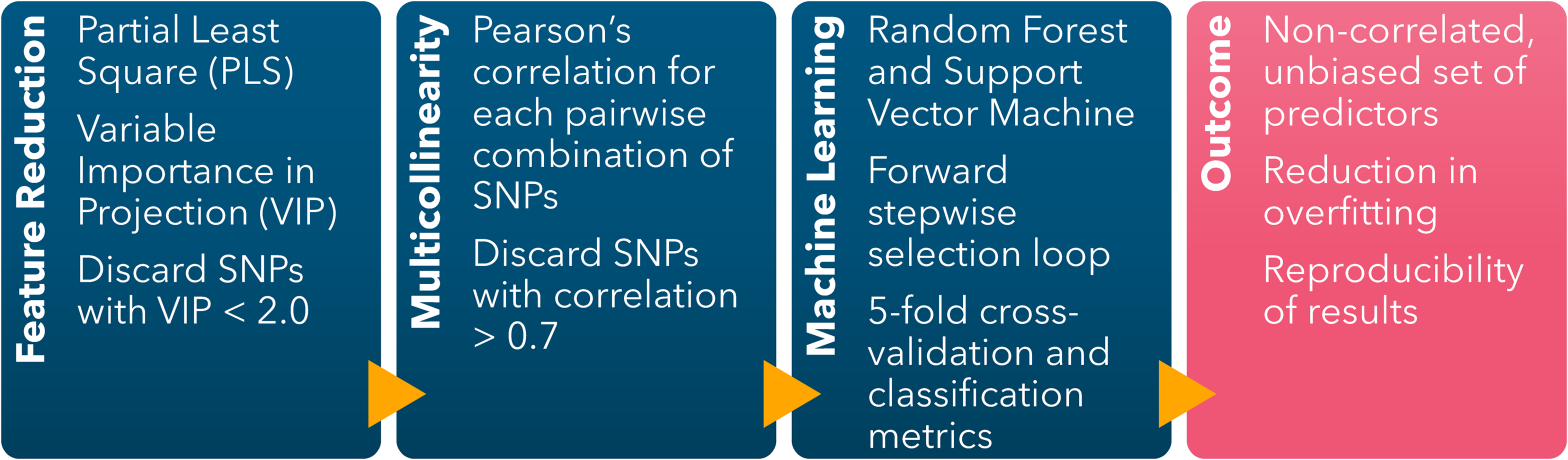
Machine learning-based GWAS pipeline scheme including feature dimension reduction (Partial Least Square), reduction of multicollinearity (Pairwise Pearson’s Correlation), and identification of sets of SNPs conferring the highest classification accuracy (Forward stepwise selection loop using Random Forest and Support Vector Machine).

The ML-GWAS pipeline to identify the combination of predictors yielding the highest prediction accuracy was implemented following the protocol first described by Canella Vieira et al. (2022c) (**Figure 1**). A Partial Least Square (PLS) (Hold, 1966) model was fitted using the off-target dicamba damage scores as the variable response and the 4,970 SNPs as predictors. The components’ coefficients were trained using a 10-fold cross-validation to achieve a low validation error. The relative importance of each predictor in the components was represented by the Variable Importance in Projection (VIP) scores. The analysis was conducted in R (R Core Team, 2023) using the package ‘*pls*’ to fit the PLS model (Mevik and Wehrens, 2007) and ‘*plsVarSel*’ to obtain the VIP scores (Mehmood et al., 2012).

The SNPs with VIP scores< 2.0 were discarded. Among the SNPs with VIP ≥ 2.0, Pearson’s correlation coefficients were calculated for each possible pairwise combination. For each iteration, if the pair-wise correlation was< 0.7, both SNPs were kept. The SNP with the lowest VIP was discarded when an absolute pairwise correlation ≥ 0.7 occurred. The loop finished after the last possible pair-wise correlation was calculated. The objective of this filtering step was to limit overfitting and multicollinearity by discarding highly correlated predictors with low relative importance to the response variable.

The non-correlated selected SNPs with VIP ≥ 2.0 were included as predictors in the Random Forest (RF) and Support Vector Machine (SVM) models in a forward stepwise selection loop to identify the combination of SNPs yielding the highest classification accuracy. The selection loop started by fitting the SNP with the highest VIP, followed by adding each SNP one at a time. The SNP yielding the highest accuracy in the preceding iteration was retained in the subsequent loop, of which the classification accuracy was calculated with an additional SNP. The loop concluded when no further improvement in the classification accuracy was observed by the addition of another SNP, thereby identifying the optimal combination of predictors. To evaluate the impact of overfitting on the prediction accuracy of both models, the loop continued despite no additional gain in classification accuracy, and classification accuracy metrics were recorded for each iteration.

Each iteration was analyzed with 5-fold cross-validation and classification accuracy metrics were recorded. The overall accuracy of each iteration was computed using eq. 1. Class accuracy is represented by the proportion of true positives (TP) and true negatives (TN) for individual classes (sum of TP, TN, false positive (FP), and false negative (FN)) (Eq. 2). Precision is described as the number of TP by the number of predicted positives (TP + FP) for individual classes (Eq. 3). Specificity is defined by the ratio of TN by TN and FP for individual classes (Eq. 4).


(1) $$\text{Overall}\;\text{Accuracy}=\;\frac{\text{No}.\text{ of}\;\text{Correct}\;\text{Classifications}}{\text{Total No}.\text{ of Samples}}\;\times 100\%$$

(2) $$\text{Class Accuracy}=\;\frac{\text{TP}+\text{TN}}{\text{TP}+\text{TN}+\text{FP}+\text{FN}}$$

(3) $$\text{Precision}=\;\frac{\text{TP}}{\text{TP}+\text{FP}}$$

(4) $$\text{Specificity}=\;\frac{\text{TN}}{\text{TN}+\text{FP}}$$

where,

TP = True Positive (correctly predicted as the positive class);
TN = True Negative (correctly predicted as the negative class);
FP = False Positive (incorrectly predicted as the positive class);
FN = False Negative (incorrectly predicted as the negative class).

RF and SVM machine learning models were used for the multi-class prediction problem. These were chosen based on their efficacy in handling data in which the number of predictors is larger than the number of observed samples, as well as a providing satisfactory balance between the variance-bias trade-off (James et al., 2013). RF is a supervised learning algorithm based on the assembly of multiple decision trees. It conducts feature selection and generates non-correlated decision trees making it feasible to include a high number of predictors in the model (Breiman, 2001). The SVM model places flexible hyperplanes among classes, being particularly useful in classification problems. The model provides flexibility to identify combinations of adjustable parameters that optimize model performance while mitigating the risk of overfitting.

The RF model was conducted using the R package ‘*randomForest*’ (Liaw and Wiener, 2002) with the square root of *p* predictors (number of predictors) randomly selected at each split. The SVM model was conducted using the R package ‘*e1071*’ (Meyer et al., 2021) with the kernel defined as ‘radial’. The optimal combination of trainable parameters was provided using the function ‘*tune*’. The final model was tunned using a grid search for the cost ranging from 0.01, 0.1, 1, 10, 100, and 1000, and gamma ranging from 0.0001, 0.001, 0.01, 0.5, and 1 (Canella Vieira et al., 2022c).

## 3 Results

### 3.1 Phenotypic distribution

**Figure 2:**
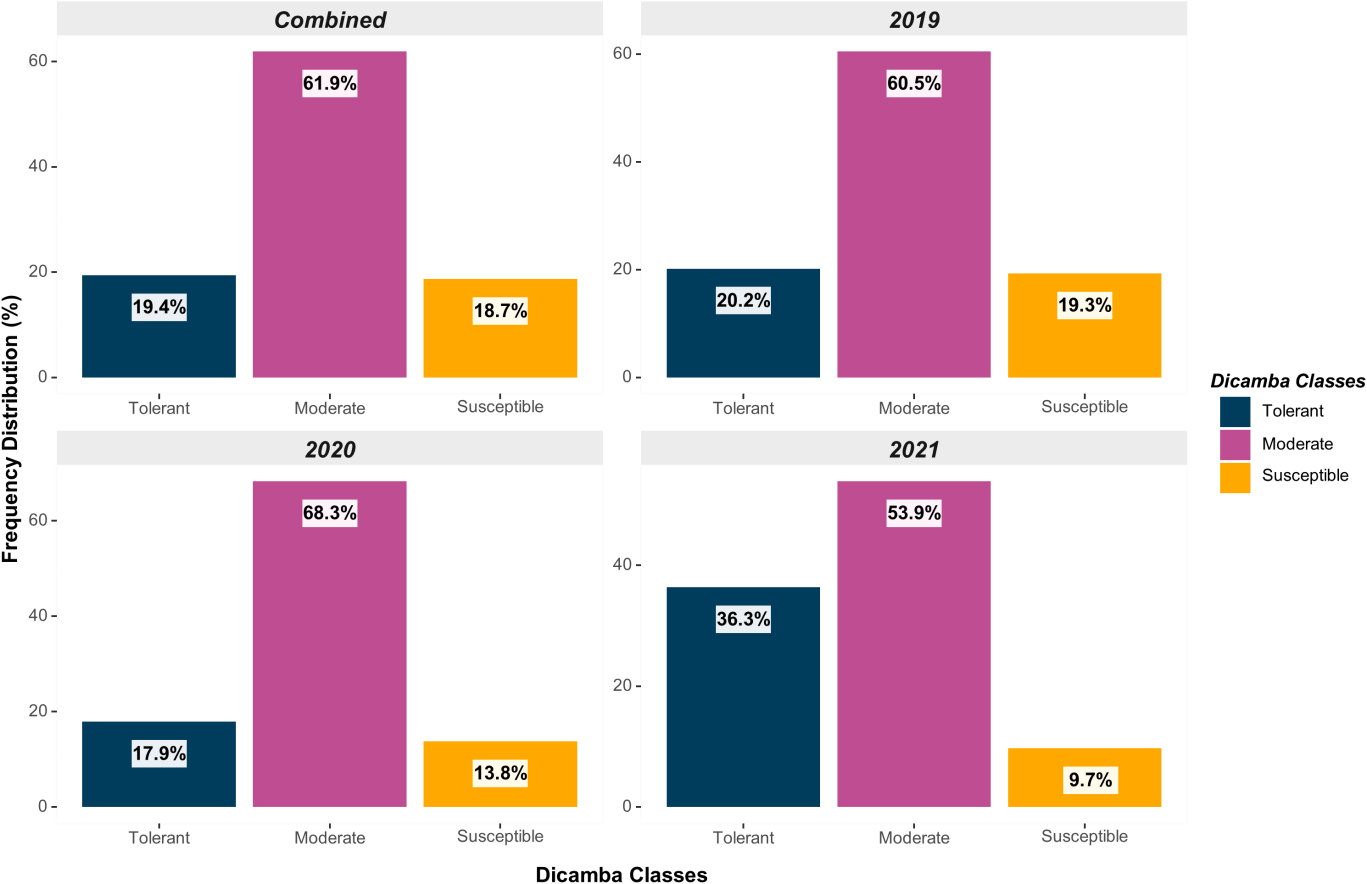
Distribution of genotypes based on off-target dicamba response (tolerant, moderate, and susceptible) within each year and across all testing environments.

Across all testing years (2019-2021), a total of 107 genotypes were classified as tolerant (19.4%), 341 as moderate (61.9%), and 103 as susceptible (18.7%) (**Figure 2**). The distribution was relatively uniform across the years, although the frequency of susceptible genotypes declined over the years as a result of the potential indirect selection of tolerant genotypes based on favorable agronomic traits and yield in environments exposed to prolonged off-target dicamba. Indirect selection has been documented in soybean for multiple traits, including off-target dicamba tolerance (Canella Vieira et al., 2022b), adaptation and maturity (Board et al., 1997), seed size (LeRoy et al., 1991), and grain yield (Board et al., 2003; Kahlon et al., 2011). Multi-environment grain yield, the impact of off-target dicamba exposure on yield, and the consistency and reliability of scores across and within environments of these genotypes have been previously reported by Canella Vieira et al. (2022b).

### 3.2 Genome-wide association results

**Figure 3:**
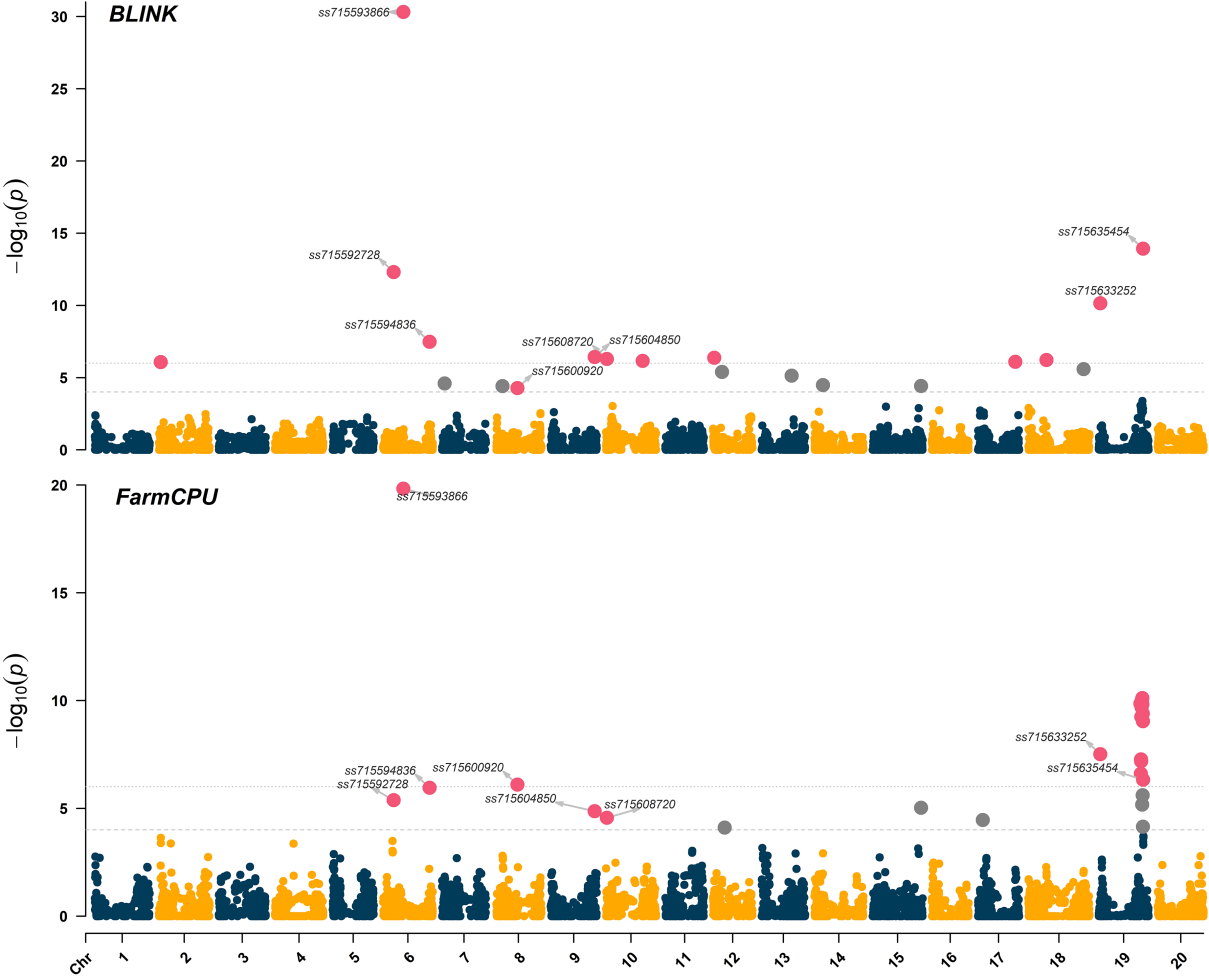
Manhattan plot highlighting in magenta the significant marker-trait associations identified using the BLINK and FarmCPU models. The threshold of marker-trait association significance was LOD > 4.0.

Significant marker-trait associations (logarithm of the odds (LOD) > 4.0) were identified using both FarmCPU and BLINK models across chromosomes 6 (LG C2), 8 (LG A2), 9 (LG K), 10 (LG O), and 19 (LG L) (**Figure 3**). The genomic regions and harboring candidate genes were reported based on the soybean assembly Williams 82 Version 2 (Genome Browser *Wm82.a2*, www.soybase.org). In chromosome 6, three separate genomic regions were detected at 10,891,060 bp, 20,739,900 bp, and 47,550,354 bp. The genomic region on chromosome 6 (10,891,060 bp) represented by the SNP *ss715592728* (minor allele frequency (MAF) of 0.33) resulted in LOD scores of 5.4 and 12.3 for the FarmCPU and BLINK models, respectively (**Table 1**). The SNP *ss715593866* (MAF of 0.47, 20,739,900 bp) had the highest LOD scores in both FarmCPU and BLINK models (19.8 and 30.3, respectively) across the entire set of SNPs. A Universal Stress Protein (*Glyma.06g209600*) has been reported within 50 kb of *ss715593866* (Genome Browser *Wm82.a2*, www.soybase.org). Lastly, *ss715594836* (MAF of 0.34, 47,550,354 bp) is co-localized with a glycosyltransferase protein (*Glyma.06g286500*) and resulted in LOD scores of 6.0 and 7.5 for the FarmCPU and BLINK models, respectively. In chromosome 8, a genomic region at 22,622,648 bp (*ss715600920*, MAF of 0.17) resulted in LOD scores of 6.1 and 4.3 for the FarmCPU and BLINK models, respectively. A gene (*Glyma.08g255800*) expressing an *S*-adenosylmethionine decarboxylase is located within 50 kb of *ss715600920*. In chromosome 9, *ss715604850* (MAF of 0.16, 48,055,288 bp) had LOD scores of 4.9 and 6.4 for the FarmCPU and BLINK models, respectively. Interestingly, an additional glycosyltransferase protein (*Glyma.09g224800*) is located within 50 kb of *ss715604850*. The genomic region identified on chromosome 10 (981,062 bp) is co-localized with the region previously reported by Canella Vieira et al. (2022a). The SNP *ss715608720* (MAF of 0.40) had LOD scores of 4.6 and 6.3 for the FarmCPU and BLINK models, respectively. Two genes with plant herbicide detoxification functions were detected within 50 kb of *ss715608720*, including *Glyma.10g010000* (glycosyltransferase protein) and *Glyma.10g010700* (oxidoreductase activity). Lastly, a novel genomic region in chromosome 19 was identified at 1,656,743 bp. The SNP *ss715633252* (MAF of 0.47) had LOD scores of 7.5 and 10.0 for the FarmCPU and BLINK models, respectively. Two ATP-binding cassette (ABC) transporter family proteins (*Glyma.19g016400* and *Glyma.19g016600*) were identified within 50kb of this SNP. A second genomic region at 45,152,186 bp of chromosome 19 was also detected. This region was previously reported by Canella Vieira et al. (2022a) and is rich in UDP-dependent glycosyltransferase genes. The SNP *ss715635454* (MAF of 0.33) had LOD scores of 6.3 and 13.9 for the FarmCPU and BLINK models, respectively. Across all significant marker-trait associations, the reported candidate genes have biological functions directly associated with the multi-phase herbicide detoxification model (Riechers et al., 2010). The genomic regions on chromosomes 6, 8, 9, and 19 identified in this study have not been previously reported as associated with off-target dicamba response and may be the focus of further investigations to understand the physiological mechanisms conferring tolerance.

**Table 1:** Summary of significant marker-trait associations identified using the BLINK and FarmCPU models including genomic position, minor allele frequency, logarithm of odds, variable importance in projection, and co-localized candidate genes.

SNP	Chr.	Position	MAF (%)^2^	LOD^3^		VIP^4^	Candidate Genes^5^	Function^5^
		(bp)^1^		BLINK	FarmCPU			
*ss715592728*	6	10,891,060	0.33	12.3	5.4	2.46		
*ss715593866*	6	20,739,900	0.47	30.3	19.8	3.16	*Glyma.06g209600*	Universal Stress Protein
*ss715594836*	6	47,550,354	0.34	7.5	6.0	3.07	*Glyma.06g286500*	Glycosyltransferase
*ss715600920*	8	22,622,648	0.17	4.3	6.1	2.30	*Glyma.08g255800*	S-adenosylmethionine decarboxylase
*ss715604850*	9	44,855,340	0.16	6.4	4.9	1.85	*Glyma.09g224800*	Glycosyltransferase
*ss715608720*	10	981,062	0.40	6.3	4.6	2.23	*Glyma.10g010000*	Glycosyltransferase
							*Glyma.10g010700*	Oxidoreductase
*ss715633252*	19	1,656,743	0.47	10.0	7.5	2.83	*Glyma.19g016400*	ABC Transporter Protein
							*Glyma.19g016600*	ABC Transporter Protein
*ss715635454*	19	45,152,186	0.33	13.9	6.3	2.27	*Glyma.19g187400*	UDP-glycosyltransferase genes

^1^Position in the genome reported as base pairs (Genome assembly version Wm82.a2). ^2^Minor allele frequency reported in percentage. ^3^LOD, the logarithm of odds calculated as the negative logarithm of the observed p-value for each model. VIP, variable importance in projection. ^5^Candidate Genes and Functions identified within a 50 kb window from the significant SNP (Genome Browser *Wm82.a2*, www.soybase.org).

### 3.3 Variable importance in projection and classification metrics

**Figure 4:**
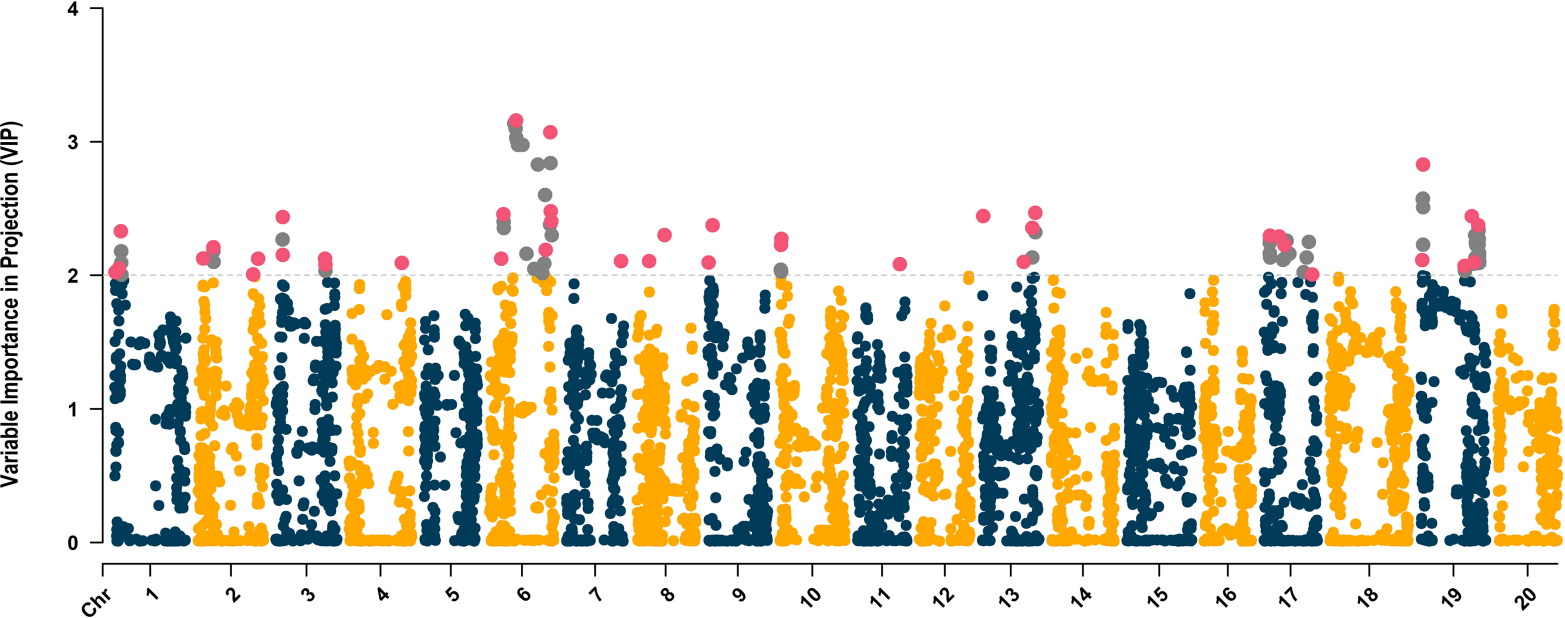
Variable Importance in Projection (VIP)-based Manhattan plot of the 4,970 SNPs. The SNPs with VIP scores higher than 2.0 are highlighted in gray, and the 41 uncorrelated SNPs selected to be used in the ML-based GWAS are colored in magenta.

The distribution of SNPs across chromosomes was relatively uniform with an average of 248 SNPs per chromosome, ranging from 190 (chromosome 17, LG D2) to 327 SNPs (chromosome 8). The average VIP score across 4,970 SNPs was 0.82, ranging from 0.01 (*ss715598194*) to 3.16 (*ss715593866*) (**Figure 4**). Within chromosomes, the average VIP score ranged from 0.59 (chromosome 8) to 1.02 (chromosome 19). The VIP metric ranks predictors (SNPs) based on their significance to the aggregate index (*D_e_*). Given the average of squared VIP scores are equal to 1.0, a threshold higher than 1.0 is employed to select features that make the most substantial contribution to *D_e_* (Chong and Jun, 2005; Cocchi et al., 2018). In scenarios where the number of independent variables significantly exceeds the number of observations and there is considerable multicollinearity, a threshold of 2.0 is suggested to filter significant predictors (Cocchi et al., 2018; Canella Vieira et al., 2022c). A total of 113 SNPs with VIP scores above 2.0 were distributed across chromosomes 1 (7 SNPs, LG D1a), 2 (6 SNPs, LG D1b), 3 (6 SNPs, LG N), 4 (1 SNP, C1), 6 (25 SNPs), 7 (1 SNP, LG M), 8 (2 SNPs), 9 (2 SNPs), 10 (4 SNPs), 11 (1 SNP, LG B1), 13 (6 SNPs, LG F), 17 (16 SNPs), and 19 (36 SNPs) (**Figure 4**). To further reduce model overfitting, SNPs with absolute values of pairwise Pearson’s correlation ≥ 0.7 were removed, resulting in 41 SNPs selected to be included in the ML algorithms. These SNPs were distributed across chromosomes 1 (3 SNPs), 2 (4 SNPs), 3 (4 SNPs), 4 (1 SNP), 6, (7 SNPs), 7 (1 SNP), 8 (2 SNPs), 9 (2 SNPs), 10 (2 SNPs), 11 (1 SNP), 13 (4 SNPs), 17 (4 SNPs), and 19 (6 SNPs) (**Figure 4**).

**Figure 5:**
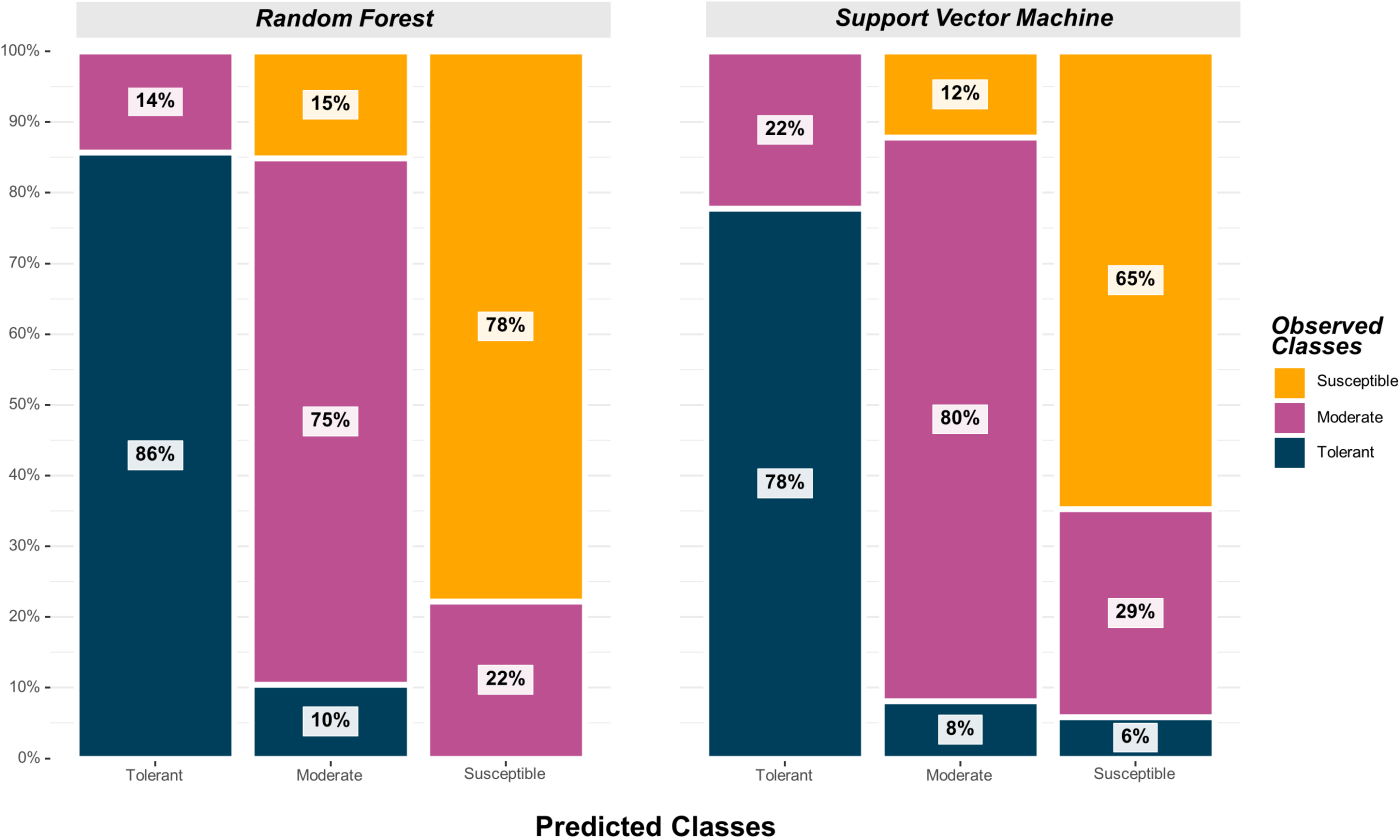
Graphical confusion matrix based on the precision of each predicted class in the Random Forest and Support Vector Machine models.

The SVM model yielded the highest overall classification accuracy (0.79) including 12 SNPs as predictors, with a noticeable reduction in overall classification accuracy with the inclusion of more SNPs (**Table 2**). The SNPs that resulted in the highest classification accuracy, sorted by order of inclusion in the model, were *ss715593866, ss715600920, ss715594836, ss715592728, ss715635403, ss715627948, ss715579081, ss715588076, ss715582179, ss715608720, ss715586851, ss715634898*, and *ss715616396*. The model including the 12 SNPs as predictors outperformed both model including only the highest VIP SNP (*ss715593866*) and the model including all 41 selected SNPs by approximately 11% (0.71 to 0.79) (**Table 2**). All classification metrics, including precision and specificity, observed equivalent improvements. The SVM model resulted in minor extreme misclassifications (observed tolerant predicted as susceptible, and vice-versa) highlighting its high suitability to be implemented in an applied soybean breeding pipeline aiming to identify genotypes tolerant to off-target dicamba (**Figure 5**). For instance, out of all tolerant predictions, 78% were observed as tolerant and 22% as moderate, while out of all susceptible predictions, 65% were observed as susceptible, 29% as moderate, and only 6% as tolerant (**Figure 5**).

**Table 2:** Summary of SVM model classification accuracy metrics based on the number of predictors.

# SNPs^1^	Overall Accuracy^2^	Tolerant			Moderate			Susceptible		
		Accuracy^3^	Precision^4^	Specificity^5^	Accuracy	Precision	Specificity	Accuracy	Precision	Specificity
1	0.71	0.50	–	1.00	0.60	0.72	0.95	0.67	0.62	0.94
2	0.71	0.50	–	1.00	0.58	0.71	0.95	0.68	0.67	0.96
3	0.72	0.55	0.50	0.98	0.62	0.73	0.93	0.68	0.67	0.96
4	0.70	0.61	0.57	0.97	0.58	0.71	0.95	0.56	0.60	0.98
5	0.72	0.63	0.56	0.96	0.65	0.76	0.89	0.67	0.57	0.93
6	0.69	0.51	0.20	0.96	0.59	0.72	0.91	0.68	0.67	0.96
7	0.68	0.61	0.57	0.97	0.60	0.73	0.86	0.63	0.47	0.91
8	0.75	0.65	0.71	0.98	0.70	0.79	0.88	0.75	0.57	0.90
9	0.74	0.65	0.73	0.99	0.69	0.78	0.88	0.72	0.52	0.89
10	0.75	0.70	0.64	0.96	0.68	0.78	0.89	0.70	0.64	0.94
11	0.77	0.74	0.71	0.99	0.70	0.78	0.93	0.67	0.62	0.94
**12**	**0.79**	**0.81**	**0.78**	**1.00**	**0.71**	**0.80**	**0.95**	**0.65**	**0.65**	**0.96**
13	0.79	0.81	0.78	1.00	0.71	0.79	0.95	0.65	0.64	0.96
14	0.77	0.81	0.77	1.00	0.69	0.78	0.93	0.62	0.55	0.94
15	0.78	0.81	0.67	1.00	0.70	0.78	0.93	0.65	0.58	0.94
16	0.78	0.78	0.76	1.00	0.70	0.78	0.93	0.67	0.62	0.94
17	0.76	0.78	0.75	1.00	0.67	0.77	0.93	0.62	0.55	0.94
18	0.76	0.75	0.77	1.00	0.67	0.77	0.93	0.65	0.58	0.94
19	0.74	0.74	0.67	0.98	0.67	0.77	0.86	0.68	0.53	0.91
20	0.75	0.78	0.69	0.99	0.67	0.77	0.89	0.64	0.50	0.92
21	0.75	0.74	0.72	0.98	0.69	0.78	0.88	0.71	0.59	0.92
22	0.74	0.70	0.70	0.97	0.68	0.78	0.85	0.73	0.58	0.91
23	0.75	0.70	0.70	0.97	0.68	0.78	0.86	0.74	0.61	0.92
24	0.75	0.70	0.70	0.97	0.69	0.78	0.88	0.74	0.65	0.93
25	0.75	0.71	0.72	0.98	0.68	0.78	0.89	0.72	0.63	0.93
26	0.76	0.71	0.72	0.98	0.70	0.79	0.89	0.74	0.65	0.93
27	0.75	0.71	0.78	0.98	0.68	0.78	0.89	0.72	0.63	0.93
28	0.76	0.71	0.78	0.98	0.70	0.79	0.89	0.74	0.65	0.93
29	0.76	0.71	0.78	0.98	0.70	0.79	0.89	0.74	0.65	0.93
30	0.76	0.71	0.78	0.98	0.70	0.79	0.89	0.74	0.65	0.93
31	0.76	0.71	0.78	0.98	0.70	0.79	0.89	0.74	0.65	0.93
32	0.77	0.71	0.76	0.99	0.70	0.79	0.91	0.74	0.65	0.93
33	0.78	0.74	0.70	0.99	0.72	0.80	0.91	0.74	0.65	0.93
34	0.75	0.71	0.75	0.98	0.69	0.78	0.88	0.74	0.61	0.92
35	0.76	0.71	0.75	0.98	0.70	0.79	0.88	0.76	0.63	0.92
36	0.74	0.71	0.75	0.99	0.68	0.78	0.84	0.74	0.52	0.88
37	0.70	0.71	0.75	0.99	0.65	0.77	0.80	0.70	0.44	0.84
38	0.70	0.71	0.74	0.99	0.65	0.77	0.80	0.70	0.44	0.84
39	0.70	0.71	0.73	0.99	0.65	0.77	0.80	0.70	0.44	0.84
40	0.71	0.68	0.75	0.98	0.66	0.77	0.82	0.71	0.48	0.87

^1^Number of SNPs included in the model in each iteration. The highest classification accuracy (0.79) was obtained with 12 SNPs as predictors, including *ss715593866, ss715600920, ss715594836, ss715592728, ss715635403, ss715627948, ss715579081, ss715588076, ss715582179, ss715608720, ss715586851, ss715634898, and ss715616396*. ^2^Overall classification accuracy calculated based on Eq. 1. ^3^Class Accuracy calculated based on Eq. 2. ^4^Precision calculated based on Eq. 3. ^5^Specificity calculated based on Eq. 4.

The bold values indicate the iteration which conferred the highest classification accuracy.

**Figure 6:**
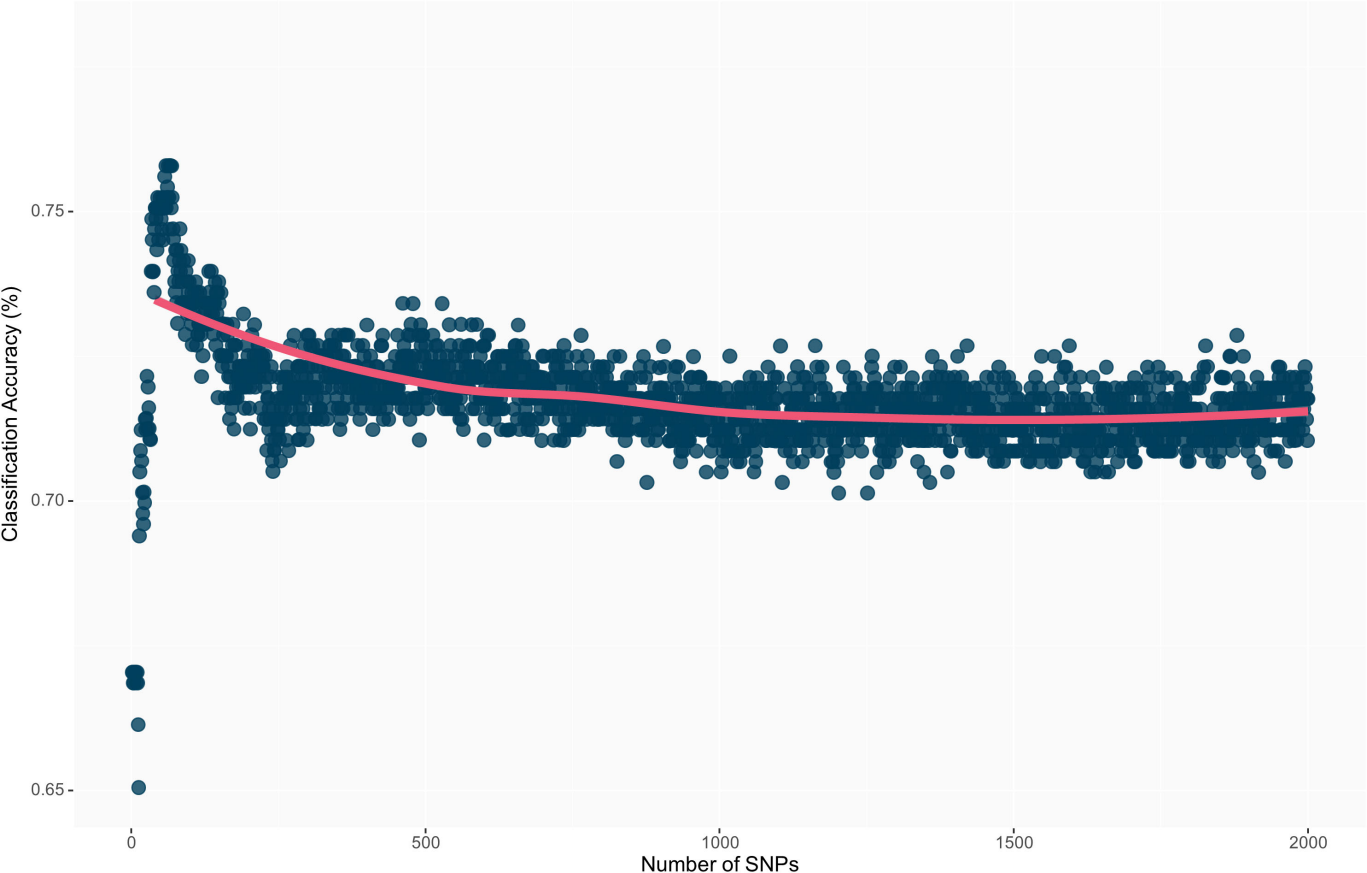
Overall prediction accuracy of each model’s iteration from 1 to 2,000 SNPs as predictors. The decrease in prediction accuracy with the increment of the number of SNPs is a result of model overfitting.

The highest overall classification accuracy (0.76) in the RF model was achieved using 17 SNPs as predictors, including *ss715593866, ss715635403, ss715588076, ss715592728, ss715600920, ss715582179, ss715633252, ss715626266, ss715583058, ss715582533, ss715610029, ss715605561, ss715605251, ss715599209, ss715627948, ss715616396, ss715595654*, and *ss715580115* (**Table 3**). Eight SNPs, including *ss715593866, ss715635403, ss715588076, ss715592728, ss715600920, ss715582179, ss715627948*, and *ss715616396* overlapped between the SVM and RF models yielding the highest overall classification accuracy. A larger increase in overall classification accuracy (17%) was observed between the baseline model including only *ss715593866* (0.65) and the model including 17 SNPs (0.76). Substantial improvements in class accuracy, precision, and specificity were also observed between the two models. The RF model also demonstrated high suitability to be implemented in real-world prediction problems. Out of all tolerant predictions, 86% were observed as tolerant and 14% as moderate, while out of all susceptible predictions, 78% were observed as susceptible and 22% as moderate (**Figure 5**). Overall, the RF model did not perform any extreme misclassifications. Similar to the SVM model, a substantial decrease in overall classification accuracy was observed with the inclusion of more predictors (**Figure 6**). The overall classification accuracy was computed for each iteration from 1 SNP to 2,000 SNPs. A pronounced negative trend was observed with the increase in SNPs, indicating the negative impact of overfitting and the importance of filtering SNPs on overall model performance (**Figure 6**).

**Table 3:** Summary of RF model classification accuracy metrics based on the number of predictors.

# SNPs	Overall Accuracy	Tolerant			Moderate			Susceptible		
		Accuracy	Precision	Specificity	Accuracy	Precision	Specificity	Accuracy	Precision	Specificity
1	0.65	0.61	0.67	0.98	0.54	0.69	0.97	0.50	–	1.00
2	0.66	0.56	0.67	0.99	0.52	0.68	0.99	0.50	–	1.00
3	0.67	0.63	0.50	0.95	0.55	0.70	0.88	0.49	0.14	0.93
4	0.67	0.63	0.56	0.96	0.59	0.72	0.93	0.56	0.60	0.98
5	0.67	0.63	0.56	0.96	0.62	0.74	0.91	0.63	0.60	0.96
6	0.69	0.68	0.75	0.98	0.65	0.75	0.91	0.61	0.46	0.92
7	0.70	0.67	0.67	0.97	0.64	0.75	0.92	0.63	0.60	0.96
8	0.70	0.64	0.63	0.97	0.61	0.73	0.88	0.64	0.54	0.93
9	0.72	0.61	0.67	0.98	0.61	0.73	0.92	0.65	0.64	0.96
10	0.73	0.61	0.67	0.98	0.63	0.74	0.93	0.65	0.64	0.96
11	0.74	0.62	0.80	0.99	0.66	0.76	0.96	0.68	0.73	0.97
12	0.73	0.62	0.80	0.99	0.65	0.75	0.96	0.69	0.80	0.98
13	0.72	0.58	0.60	0.98	0.61	0.73	0.92	0.68	0.67	0.96
14	0.74	0.63	0.71	1.00	0.63	0.74	0.93	0.67	0.62	0.94
15	0.71	0.59	0.74	1.00	0.61	0.73	0.91	0.66	0.66	0.92
16	0.75	0.69	0.75	1.00	0.66	0.74	0.93	0.67	0.70	0.94
**17**	**0.76**	**0.68**	**0.86**	**0.99**	**0.67**	**0.75**	**0.93**	**0.70**	**0.78**	**0.96**
18	0.75	0.65	0.83	0.99	0.65	0.75	0.93	0.68	0.76	0.96
19	0.75	0.68	0.75	0.98	0.67	0.76	0.92	0.70	0.72	0.96
20	0.75	0.65	0.83	0.99	0.65	0.75	0.93	0.68	0.67	0.96
21	0.72	0.61	0.67	0.98	0.61	0.73	0.92	0.65	0.64	0.96
22	0.72	0.61	0.67	0.98	0.61	0.73	0.92	0.65	0.64	0.96
23	0.71	0.61	0.67	0.98	0.60	0.72	0.92	0.63	0.60	0.96
24	0.70	0.61	0.67	0.98	0.60	0.73	0.89	0.64	0.54	0.93
25	0.70	0.61	0.67	0.98	0.60	0.73	0.89	0.64	0.54	0.93
26	0.72	0.65	0.71	0.98	0.62	0.74	0.91	0.65	0.58	0.94
27	0.70	0.61	0.67	0.98	0.61	0.73	0.88	0.66	0.53	0.92
28	0.74	0.68	0.86	0.99	0.64	0.75	0.92	0.65	0.58	0.94
29	0.71	0.61	0.67	0.98	0.61	0.73	0.89	0.67	0.57	0.93
30	0.70	0.65	0.83	0.99	0.60	0.73	0.89	0.61	0.46	0.92
31	0.70	0.61	0.67	0.98	0.61	0.73	0.88	0.66	0.53	0.92
32	0.71	0.65	0.83	0.99	0.62	0.74	0.88	0.66	0.50	0.91
33	0.69	0.62	0.80	0.99	0.59	0.72	0.88	0.63	0.47	0.91
34	0.69	0.65	0.83	0.99	0.59	0.72	0.88	0.61	0.43	0.91
35	0.71	0.65	0.71	0.98	0.62	0.74	0.88	0.66	0.53	0.92
36	0.70	0.65	0.83	0.99	0.61	0.74	0.86	0.65	0.47	0.90
37	0.70	0.62	0.80	0.99	0.60	0.73	0.89	0.64	0.50	0.92
38	0.67	0.59	0.75	0.99	0.57	0.71	0.86	0.63	0.44	0.90
39	0.69	0.65	0.83	0.99	0.60	0.73	0.86	0.63	0.44	0.90
40	0.69	0.65	0.83	0.99	0.59	0.72	0.88	0.61	0.43	0.91

^1^Number of SNPs included in the model in each iteration. The highest classification accuracy (0.76) was obtained with 17 SNPs as predictors, including *ss715593866, ss715635403, ss715588076, ss715592728, ss715600920, ss715582179, ss715633252, ss715626266, ss715583058, ss715582533, ss715610029, ss715605561, ss715605251, ss715599209, ss715627948, ss715616396, ss715595654, and ss715580115*. ^2^Overall classification accuracy calculated based on Eq. 1. ^3^Class Accuracy calculated based on Eq. 2. ^4^Precision calculated based on Eq. 3. ^5^Specificity calculated based on Eq. 4.

The bold values indicate the iteration which conferred the highest classification accuracy.

## 4 Discussion

The development of DT soybean and cotton (*Gossypium hirsutum* L.) was a major biotechnology breakthrough grounded on diversifying strategies of herbicide-based weed management as well as overcoming weeds resistant to glyphosate at a time when GT was the only genetically-engineered herbicide tolerance system available (Behrens et al., 2007). The discovery of metabolism of dicamba to 3,6-dichlorosalicylic acid (DCSA) (Taraban et al., 1993; Fogarty and Tuovinen, 1995; Herman et al., 2005), a compound without herbicidal activity, by soil bacteria under both aerobic and anaerobic conditions led to the development of DT plants by inserting the bacterial gene *dicamba monooxygenase* (*DMO*) from *Pseudomonas maltophilia* (Strain DI-6) (Behrens et al., 2007). Genetically-engineered plants expressing the enzyme dicamba *O*-demethylase convert dicamba to DCSA before it accumulates to phytotoxic levels (Herman et al., 2005; Behrens et al., 2007; Wang et al., 2016). In the United States, DT soybean seeds were first commercialized in 2016 and were rapidly adopted on nearly 22.3 million hectares (Tindall et al., 2021).

The goal of this study was to detect genomic regions related to various responses to prolonged off-target dicamba exposure in a population consisting of advanced soybean breeding lines. A total of 551 non-DT advanced breeding lines derived from 232 unique bi-parental populations were grown in environments surrounded by DT soybean and cotton growing systems, thus being exposed to prolonged unintentional off-target dicamba. Although each testing environment showed homogenous off-target dicamba distribution (Canella Vieira et al., 2022b), one limitation of this study was the lack of precise data regarding the dosage of dicamba received by each experimental plot during specific growth stages and during the growing season. The various sources and dosages of dicamba combined with fluctuating environmental factors make it unfeasible to accurately quantify the exposure in a large-scale field study (Kniss, 2018; Canella Vieira et al., 2022b). Experiments in controlled environments with specific pre-determined dosages should be conducted to further investigate thresholds at which the identified genomic regions can maintain the observed responses.

A total of eight genomic regions related to various responses to off-target dicamba were identified across chromosomes 6 (3), 8 (1), 9 (1), 10 (1), and 19 (2). Interestingly, several candidate genes co-localized with significant SNPs have been reported to have biological functions directly related to proteins participating in the three phases of herbicide detoxification in plants (Riechers et al., 2010). Thus, it can be hypothesized that non-DT soybean genotypes with tolerance response to off-target dicamba may have the capability to more rapidly detoxify low doses of the herbicide compared to sensitive genotypes. For instance, the gene *Glyma.06g209600* is located within 50 kb of *ss715593866* (LOD scores of 19.8 and 30.3 in the FarmCPU and BLINK models, respectively). This gene has been reported to be a Universal Stress Protein with adenine nucleotide alpha hydrolase function. Phase I of herbicide detoxification usually introduces a reactive functional group for the subsequent metabolism and detoxification through oxidation or hydrolysis by cytochrome P450s or carboxylesterases, respectively (Kreuz et al., 1996; Barrett, 2000). Although the genetic architecture of tolerance is complex and regulated by multiple small and large effect loci, *ss715593866* is a major effect SNP and resulted in high classification accuracies in both RF and SVM when included as the sole predictor. Therefore, further investigation of the role and effect of *ss715593866* could better explain the physiological mechanisms associated with tolerance to off-target dicamba in soybean.

*Glyma.06g286500* is a candidate gene located within 50 kb of *ss715594836* (LOD scores of 6.0 and 7.5 in the FarmCPU and BLINK models, respectively) with glycosyltransferase-related functions. Phase II of herbicide detoxification involves conjugation reactions of herbicides with reduced glutathione [catalyzed by glutathione *S*-transferases (GST)] or glucose (catalyzed by UDP-dependent glycosyltransferases) (Riechers et al., 2010). In chromosome 8, the candidate gene *Glyma.08g255800* located within 50kb of *ss715600920* (LOD scores of 6.1 and 4.3 for the FarmCPU and BLINK models, respectively) expresses an *S*-adenosylmethionine decarboxylase. This enzyme is key in the biosynthesis of polyamines (Majumdar et al., 2017). Although the precise role of *S*-adenosylmethionine decarboxylase in plants is still unknown, its up-regulation has been reported in response to many abiotic stressors including salt (Hao et al., 2005), drought (Urano et al., 2003; Alcázar et al., 2006), temperature (Hao et al., 2005; Cheng et al., 2009), and oxidative stress (Moschou et al., 2008). A consequence of exposure to auxinic herbicides is the rapid increase in ethylene production by initiating 1-aminocyclopropane-1-carboxylic acid synthase and biosynthesis of abscisic acid (Hansen and Grossmann, 2000; Grossmann et al., 2001; Kraft et al., 2007). This reduces transpiration, carbon dioxide assimilation, starch formation, and a substantial accumulation of reactive oxygen species, which leads to chloroplast damage, membrane destruction, and ultimately tissue damage and cell death (Kraft et al., 2007; Grossmann, 2010).

Similar to *Glyma.06g286500*, the candidate genes *Glyma.09g224800* (co-localized with *ss715604850*, LOD scores of 4.9 and 6.4 for the FarmCPU and BLINK models, respectively) and *Glyma.10g010000* (co-localized with *ss715608720*, LOD scores of 4.6 and 6.3 for the FarmCPU and BLINK model, respectively) have glycosyltransferase-related functions which are associated with conjugation reactions of phase II of herbicide detoxification (Riechers et al., 2010). Within the same genomic region of chromosome 10, *ss715608720* is also co-localized with *Glyma.10g010700*, a candidate gene involved in oxidoreductase activity. The expression of oxydoreductase enzymes acts as a signal to the subsequential expression of GST, cytochrome P450 monooxygenases, and other proteins involved in herbicide detoxification (Zhang et al., 2007; Riechers et al., 2010). This genomic region was previously reported, and the candidate gene *Glyma10g01700*, which encodes a multidrug resistance protein (MRP), was co-localized with the significant SNP *ss715605561* (Canella Vieira et al., 2022a). On chromosome 19, a genomic region around 1,650,000 bp (*ss715633252*, LOD scores of 7.5 and 10.0 for the FarmCPU and BLINK models, respectively) harbors two candidate genes (*Glyma.19g016400* and *Glyma.19g016600*) that belong to the ABC transporter family. Herbicide conjugates from phase II are transported into the vacuole of plant cells by transporters, concluding phase III of herbicide detoxification (Riechers et al., 2010). Another genomic region on chromosome 19 around 45,000,000 bp was detected and previously reported by Canella Vieira et al. (2022a). This genomic region contains several UDP-glycosyltransferase genes which are necessary for phase II reactions of herbicide detoxification (Canella Vieira et al., 2022a).

One of the main challenges in analyzing high-dimensional genomic data is the presence of multicollinearity and excessive noise among predictors, which often leads to a substantial detection of false-positive associations in GWAS (Ishwaran et al., 2010; Chen and Ishwaran, 2012; Canella Vieira et al., 2022c). Given the substantial imbalance between the number of predictors (SNPs) and observations, traditional GWAS models frequently face the risk of overfitting. In this scenario, the model overly captures unintended noise in the training set, yielding low reproducibility on the testing set (Austin and Steyerberg, 2015; Ying, 2019). An approach to avoid overfitting and improve model reproducibility and cost-effectiveness is feature selection, which is the process of selecting relevant predictors from the original predictors set (Akarachantachote et al., 2014). In this study, a supervised feature dimension reduction based on VIP scores initially selected predictors with high importance toward the aggregate index (*D_e_*). This was followed by a pair-wise correlation filtering step, resulting in a subset of important, uncorrelated SNPs. In both RF and SVM models, a pronounced decrease in prediction accuracy was observed with the increment of SNPs as predictors. Therefore, identifying fewer but relevant predictors (i.e. feature selection) yielded higher prediction accuracies as compared to fitting the model with the highest number of predictors available. Equivalent results were observed by Canella Vieira et al. (2022c) when implementing a similar methodology to predict soybean resistance to southern root-knot nematode (*Meloidogyne incognita* (Kofold & White) Chitwood). In their study, a more pronounced decrease in prediction accuracy as a consequence of overfitting was observed. In addition, a lower number of predictors was needed to achieve the highest prediction accuracy, which could be explained by the qualitative nature of the phenotype. In this study, although the tolerance to off-target dicamba is substantially more complex and quantitative than resistance to southern root-knot nematode, less than 0.5% of total predictors were needed to achieve the highest prediction accuracy in both RF (17 out of 4,970 SNPs) and SVM (12 out of 4,970 SNPs) models. Singer et al. (2022) observed decreased prediction accuracies of proteinogenic methionine content in soybean seeds as a consequence of overfitting. The study reported a nearly 3-fold increase in prediction accuracy by using a subset of SNPs significantly associated with the phenotype as opposed to fitting the models with the entire set of 35,570 SNP (Singer et al., 2022). Therefore, the combination of feature selection and predictive classification algorithms may provide high accuracies in the identification and selection of genotypes with desirable phenotypes for both qualitative and quantitative traits. Further validations including traits with higher genetic complexity such as grain yield are needed and can broaden the application of genomic data toward breeding decisions in a cultivar development pipeline.

Both RF and SVM models yielded high classification accuracies using the best combination of predictors (0.76 and 0.79, respectively). Both prediction models resulted in high precision, meaning that minimal extreme misclassifications (observed tolerant predicted as susceptible, and vice-versa) were observed. Using a nearly identical panel of soybean breeding lines, Canella Vieira et al. (2022b) reported that visual assessment of off-target dicamba tolerance is directly associated with seed yield under prolonged off-target dicamba exposure. On average, a yield penalty of 8.8% (confidence interval of 7.0 to 10.6%) was observed for each unit increase in damage score on a similar 1-4 scale (Canella Vieira et al., 2022b). Therefore, the identification and development of non-DT soybean genotypes with superior tolerance to off-target dicamba can help sustain the production of non-DT herbicide-tolerance systems, which currently represent nearly 14.2 million hectares. In addition, natural tolerance may improve the sustainability of niche markets for food-graded non-GMO soybean. Genomic prediction models, such as those reported in this study, can significantly speed up the identification of genotypes with superior tolerance to off-target dicamba. The understanding of the genetics and physiological mechanisms underlying the differential responses to off-target dicamba is critical to support soybean breeding programs focusing on the development of non-DT soybean genotypes with superior tolerance to off-target dicamba.

## 5 Conclusions

The widespread adoption of DT crops resulted in numerous events involving off-target dicamba damage to non-DT vegetation. Environmental conditions that exacerbate the likelihood of off-target movement are often observed in soybean-producing regions during the growing season, hence the reports of damage in most states where the over-the-top use of dicamba is authorized. Soybean is highly sensitive to dicamba exposure, critically compromising the yield and quality of non-DT genetically engineered and non-GMO growing systems. In this study, two genomic regions conferring tolerance to off-target dicamba were confirmed from previous studies, and six novel regions were identified. The genetic architecture of tolerance is complex and regulated by multiple small and large effect loci. However, *ss715593866* is a major effect SNP and resulted in high classification accuracies in both RF and SVM when included as the sole predictor. Candidate genes with biological functions associated with herbicide detoxification in plants were co-localized with significant minor and major effect SNPs. These genes need to be further confirmed through gene-editing and controlled-environment experiments. Non-DT genotypes with tolerance were previously shown to yield significantly more than non-DT susceptible genotypes under prolonged off-target dicamba exposure. Accurate genomic prediction models have been proposed and can be implemented in soybean breeding programs to speed up the identification and development of non-DT genotypes tolerant to off-target dicamba. In addition, the negative impacts of overfitting toward model performance were reported and may guide the application of genomic prediction models.

## Data availability statement

The original contributions presented in the study are included in the article/supplementary files, further inquiries can be directed to the corresponding author.

## Author contributions

CV, DJ, JFZ, BD, DR, HN, and GS contributed to the conception and design of the study. CV, BD, DR, and GS contributed to the funding resources of the study. CV contributed to the collection of data used in this study. CV and JZ contributed to the statistical analysis of this study. CV wrote the first draft of the manuscript. All authors contributed to the article and approved the submitted version.
